# NGS coverage accurately predicts *MET* and *HER2* (*ERBB2*) gene amplifications in a real-world non-small cell lung cancer cohort

**DOI:** 10.3389/fonc.2025.1618509

**Published:** 2025-07-29

**Authors:** Adam Kowalewski, Janna Siemanowski-Hrach, Thomas Stehle, Jan Rehker, Udo Siebolts, Sabine Merkelbach-Bruse, Carina Heydt

**Affiliations:** ^1^ Faculty of Medicine and University Hospital Cologne, Institute of Pathology, University of Cologne, Cologne, Germany; ^2^ Faculty of Medicine, Bydgoszcz University of Science and Technology, Bydgoszcz, Poland; ^3^ Department of Tumor Pathology, Oncology Centre Prof. Franciszek Łukaszczyk Memorial Hospital, Bydgoszcz, Poland; ^4^ Faculty of Medicine and University Hospital Cologne, Institute for Neuropathology, University of Cologne, Cologne, Germany

**Keywords:** NGS, coverage, amplification, met, HER2, lung, cancer, NSCLC

## Abstract

**Background:**

Fluorescence *in situ* hybridization (FISH) is the current standard for detecting gene amplifications, yet its low throughput and practical constraints call for alternative methods. This study evaluates next-generation sequencing (NGS) as a potential tool for accurately predicting gene amplifications.

**Methods:**

We analyzed 66 primary non-small cell lung cancer (NSCLC) samples, tested by both NGS and FISH. FISH was conducted to detect gene amplifications in *MET* in 26 samples, in *HER2* (*ERBB2)* in 21 samples, in *PIK3CA* in 9 samples, and *KRAS* in 9 samples, with one tumor tested for both *MET* and *ERBB2*. NGS fold changes, reflected by gene coverage, were calculated as the ratio of the highest gene-specific coverage to the mean coverage across all genes.

**Results:**

Amplification was detected in 46 (68.7%) samples. NGS fold changes correlated strongly with FISH Gene/CEN ratios (Spearman’s ρ = 0.720, p < 0.001) and gene copy number per cell (Spearman’s ρ = 0.847, p < 0.001). Among FISH-negative cases, NGS fold change ranged from 0.57 to 1.95, while in FISH-positive cases, it ranged from 2.11 to 25.08.

**Conclusion:**

NGS fold changes demonstrate significant correlation with FISH metrics, supporting NGS as a promising marker for gene amplification. A fold change cutoff of 2.0 effectively distinguishes amplified from non-amplified cases, with NGS achieving a high degree of predictive reliability across the tested genes.

## 1 Introduction

In cancer diagnostics, there is a growing need for comprehensive solutions that can streamline and enhance the accuracy of detecting genetic abnormalities critical for treatment decisions. While next-generation sequencing (NGS) is increasingly being integrated into routine diagnostics, fluorescence *in situ* hybridization (FISH) continues to hold its status as the gold standard for detecting gene amplifications (1, 2). FISH provides a high-resolution view of chromosomal abnormalities, making it indispensable for validating copy number variations (CNVs). However, its use is constrained by lower throughput, limited amount of tumor material and the necessity for targeted probes, as well as observer-dependent evaluations that can introduce variability (3, 4).

In contrast, NGS offers high-throughput capabilities and the potential to quantify various biomarkers, including gene copy number (GCN) variations, small variant detection, microsatellite instability, and tumor mutational burden. This versatility makes NGS particularly advantageous, as it allows for the simultaneous analysis of multiple biomarkers in a single assay. This is especially practical given the limited amount of tumor tissue available from each patient, which can make performing separate assays for each biomarker challenging or sometimes unfeasible (5, 6). Moreover, conducting a single comprehensive assay can be more cost-effective and result in a shorter overall turnaround time (7, 8). Despite these advantages, the reliability of NGS in accurately identifying CNVs, particularly gene amplifications, remains under rigorous investigation (9, 10). This study aims to explore, whether elevations in NGS coverage can serve as reliable predictors for gene amplifications by directly comparing NGS results with those obtained through FISH.

## 2 Materials and methods

### 2.1 Sample collection and preparation

Diagnostic samples were obtained from the repository of the Institute of Pathology at the University Hospital Cologne, Germany. Formalin-fixed and paraffin-embedded (FFPE) tissue samples were obtained as part of routine clinical care under approved ethical protocols complied with the Ethics Committee of the Medical Faculty of the University of Cologne. The Ethics Committee waved the need for ethical approval for this study. Sixty-six primary non-small cell lung cancer (NSCLC) samples were included in this study, with cases initially testing negative by NGS intentionally selected to ensure thorough comparison with FISH results. Sample selection was based on availability and prior evidence suggesting particular gene amplifications. FISH analysis was performed for the amplification of the Mesenchymal Epithelial Transition (*MET*) gene on 26 samples, the Human Epidermal Growth Factor Receptor 2 (*ERBB2*) gene on 21 samples, the Phosphatidylinositol-4,5-Bisphosphate 3-Kinase Catalytic Subunit Alpha (*PIK3CA*) gene on 9 samples, and the Kirsten Rat Sarcoma Viral Oncogene Homolog (*KRAS*) gene on 9 samples. One tumor was tested for both *MET* and *ERBB2* gene amplification. In the NGS analysis, copy number gains for each gene were determined by quantifying coverage increases as fold changes. For a given target gene, the fold change was calculated by dividing the highest observed coverage among the target regions of that gene by the mean coverage across all genes in the sequencing panel.

NGS provided data on targeted regions in 27 different genes (**Supplementary Table S1**) regarding mean coverage (of all genes together) and range of coverage for each specific gene covered. The following sequences of interest were covered by our NGS panel: *MET* (exons 14 (+intron 13 and intron14), 16–19; reference sequence: NM_001127500), *ERBB2* (exons 8, 19, 20; reference sequence: NM_004448), *PIK3CA* (exons 8, 10, 21; reference sequence: NM_006218), and *KRAS* (exons 11, 15; reference sequence: NM_033360). Parallel validation with FISH was performed on all samples to verify the NGS findings.

### 2.2 Custom hybrid-capture-based sequencing assays

All samples underwent formalin fixation and paraffin embedding according to standard protocols (11). Sections of 10 µm thickness were sliced from the FFPE tissue blocks and subsequently deparaffinized. Tumor regions were macrodissected from unstained slides, using a marked hematoxylin-eosin (H&E) stained slide for reference. All slides were marked by an experienced pathologist. The samples were digested overnight with proteinase K, and DNA extraction was performed using the Maxwell RSC FFPE Plus DNA Kit (Promega, Mannheim, Germany) on the Maxwell RSC (Promega), following the manufacturer’s guidelines.

For sequencing, the DNA content was first measured using a Tecan Infinite 200 microplate reader (Tecan, Maennedorf, Switzerland) and the QuantiFluor ONE dsDNA Kit (Promega). Genomic DNA was fragmented by enzymatic fragmentation to a suitable size range (e.g. 150–200 bp) (Twist Bioscience, South San Francisco, California, United States). The fragmented DNA was then subjected to end-repair, adenylation, and ligation with sequencing adapters (Twist Library Preparation EF Kit).

A hybrid-capture enrichment protocol was employed to selectively capture the target regions of interest. Biotinylated probes complementary to the target sequences (Twist Bioscience NGS Target Enrichment Solutions with a panel of 27 genes relevant for lung cancer therapy) were hybridized to the fragmented DNA. The hybridized DNA-probe complexes were captured using streptavidin-coated magnetic beads (Dynabeads MyOne Streptavidin T1; Thermo Fisher Scientific, Waltham, Massachusetts, United States). Unbound DNA was washed away, and the captured DNA was eluted from the beads.

The enriched target DNA was then PCR-amplified using library-specific primers (KAPA HiFi HotStart ReadyMix; Roche, Basel, Switzerland) to prepare the sequencing library. The resulting library was quantified, diluted, and pooled in equal amounts. Finally, the constructed libraries were sequenced on a high-throughput sequencing platform (NextSeq 550 System; Illumina or NovaSeq; Illumina, San Diego, California, United States) using the appropriate sequencing reagent kit (NextSeq 500/550 High Output Kit v2.5; Illumina or NovaSeq 6000 SP (300c) Reagent Kit v1.5; Illumina) following the manufacturer’s recommendations. Subsequent data analysis was done using an in-house pipeline. Employing fg-bio tools (v. 2.2.2) (https://fulcrumgenomics.github.io/fgbio/), alignment steps with bwa mem (v. 0.7.17) (31) included read deduplication to exclude PCR related amplification artifacts. From GATK (v. 4.1.9.0), the CollectHsMetrics tool was used to determine coverage (12).

### 2.3 Fluorescence *In situ* hybridization

FISH analysis was performed on 4 µm thick slides prepared from the same FFPE blocks used for NGS. Probes specific to the regions of interest were obtained from ZytoVision. The procedure followed the manufacturer’s instructions, with hybridization conditions optimized for each probe set. Fluorescence signals were detected using a Microscope KP-PLUS slides (Klinipath, Duiven, Netherlands). For each gene, tissue slides were hybridized overnight with the respective Zyto-Light SPEC Dual Color Probe (ZytoVision, Bremerhaven, Germany) — *MET*/CEN7, *ERBB2*/CEN17, *PIK3CA*/CEN3, and *KRAS*/CEN12. Twenty contiguous tumor cell nuclei were individually evaluated to calculate the gene/centromere (CEN) ratio and the average GCN per cell. The classification criteria for *MET*, *ERBB2*, *PIK3CA*, and *KRAS* amplifications were adapted from previously published studies (13–18). Signals were manually counted in cells with ≤15 copies and estimated using clusters for 15 and more gene copies (10, 14).

#### 2.3.1 MET

High-level amplification was defined by a *MET*/CEN7 ratio ≥2.0 or an average *MET* GCN per cell ≥6.0, a threshold based on common practice in FISH analysis for gene amplification, as exemplified by Schildhaus in lung cancer (14). Intermediate-level GCN gain was identified when ≥50% of cells contained ≥5 *MET* signals, while low-level GCN gain was defined as ≥40% of tumor cells showing ≥4 *MET* signals as previously published. All other tumors were classified as negative. In cases of low or intermediate results, an additional forty cells from different areas were evaluated.

#### 2.3.2 ERBB2

Tumors were classified as positive for amplification if the *ERBB2*/CEN17 ratio was ≥2.0 or the average *ERBB2* GCN per cell was ≥6.0, following the American Society of Clinical Oncology/College of American Pathologists (ASCO/CAP) guidelines for HER2 testing in breast cancer (19) All other tumors were considered negative. For negative results, another forty cells from different areas were assessed.

#### 2.3.3 PIK3CA and KRAS

For *PIK3CA* and *KRAS*, positive amplification was defined by a Gene/CEN ratio ≥2.0 or an average GCN per cell ≥6.0. In the absence of specific published guidelines for these genes, this threshold was selected to align with the standard convention in FISH analysis for gene amplification, ensuring consistency with the criteria used for *ERBB2* and *MET*. Tumors not meeting these criteria were classified as negative.

### 2.4 Statistical analysis

Descriptive statistics were used to summarize the data, including mean and range for NGS fold changes, FISH Gene/CEN ratios, and average GCN per cell. The relationship between NGS fold change and FISH Gene/CEN ratios as well as average GCN per cell was evaluated using Spearman Correlation to assess the strength and direction of the association. Linear regression analyses were conducted to model the relationship between NGS fold changes and both FISH Gene/CEN ratios and average GCN per cell, providing insights into the predictive value of NGS data for FISH-determined gene amplifications. Both Spearman Correlation and Linear regression analyses were performed using the online tool available at www.socscistatistics.com.

## 3 Results

**Figure 1:**
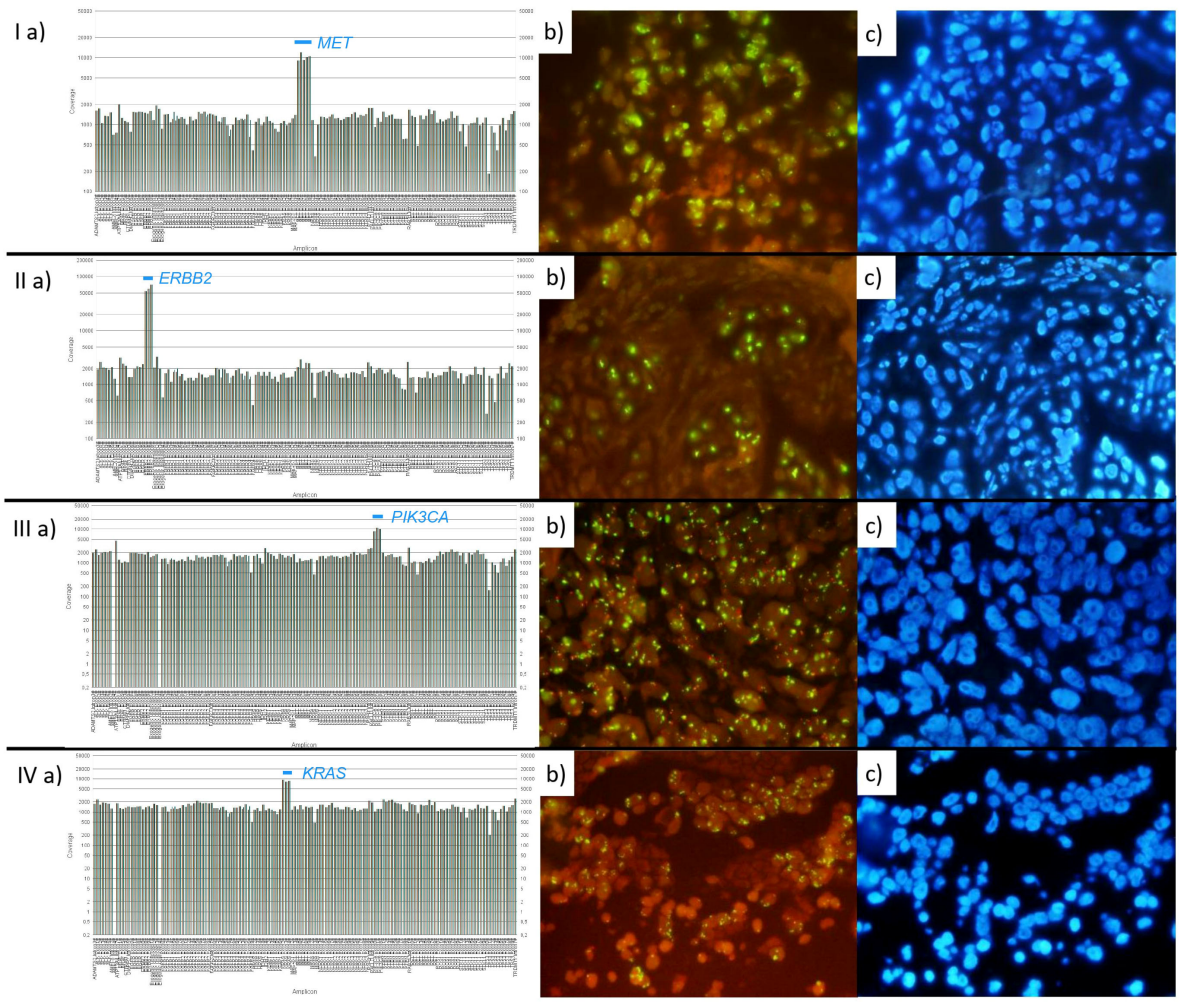
Representative cases of each gene group with elevated NGS coverage and corresponding FISH (probes and DAPI). On FISH images, gene signals are green and centromeres of reference chromosomes are marked in red. I: Case with amplified *MET*. **(a)** NGS: Elevated coverage of *MET* (8.45 fold change), **(b, c)** FISH: Gene/CEN ratio: 5.43, Copies/cell ratio: 30.95. II: Case with amplified *ERBB2*. **(a)** NGS: Elevated coverage of *ERBB2* (25.08 fold change), **(b, c)** FISH: Gene/CEN ratio: 8.59, Copies/cell ratio: 17.6. III: Case with amplified *PIK3CA*. **(a)** NGS: Elevated coverage of *PIK3CA* (6.59 fold change), **(b, c)** FISH: Gene/CEN ratio: 9.74, Copies/cell ratio: 27.75. IV: Case with amplified *KRAS*. **(a)** NGS: Elevated coverage of *KRAS* (6.04 fold change), **(b, c)** FISH: Gene/CEN ratio: 18.2, Copies/cell ratio: 21.85.

The summarized NGS and FISH results are presented in **Supplementary Table S2**. Representative cases for each gene, showing the elevation in NGS coverage along with corresponding FISH images, are depicted in **Figure 1**.

### 3.1 NGS analysis

Tumor cell content (TCC), as determined through histopathological assessment of H&E-stained slides in our cohort ranged from 10% to 80%, providing suitable conditions for NGS analysis (20). Mean NGS coverage was highest in the *MET* group at 2996.3 reads and lowest in the *KRAS* group at 209.6 reads. Fold changes in coverage varied across groups, with *MET* ranging from 1.29 to 8.45, *ERBB2* from 0.57 to 25.08, *PIK3CA* from 2.11 to 6.59, and *KRAS* from 2.32 to 6.04.

### 3.2 FISH analysis

Amplification was identified in 46 samples (68.7%), including 17 cases of *MET*, 11 of *ERBB2*, and all cases of *PIK3CA* and *KRAS*. With the exception of four MET cases (two with high-level and two with low-level amplification), all FISH-positive cases in our cohort showed positivity in both Gene/CEN ratio and GCNs per cell. In the *MET* group, the Gene/CEN ratio varied from 1.02 to 9.46, while in the *ERBB2* group, it ranged from 0.86 to 8.59. The *PIK3CA* group showed ratios between 2.9 and 11.29, and the *KRAS* group had the highest ratios, from 4.39 to 18.2. Correspondingly, GCNs per cell spanned from 2.65 to 30.95 in the *MET* group, 2.55 to 28.7 in the *ERBB2* group, 8.55 to 28.35 in the *PIK3CA* group, and 6.8 to 23.95 in the *KRAS* group.

### 3.3 Correlation analysis

**Figure 2:**
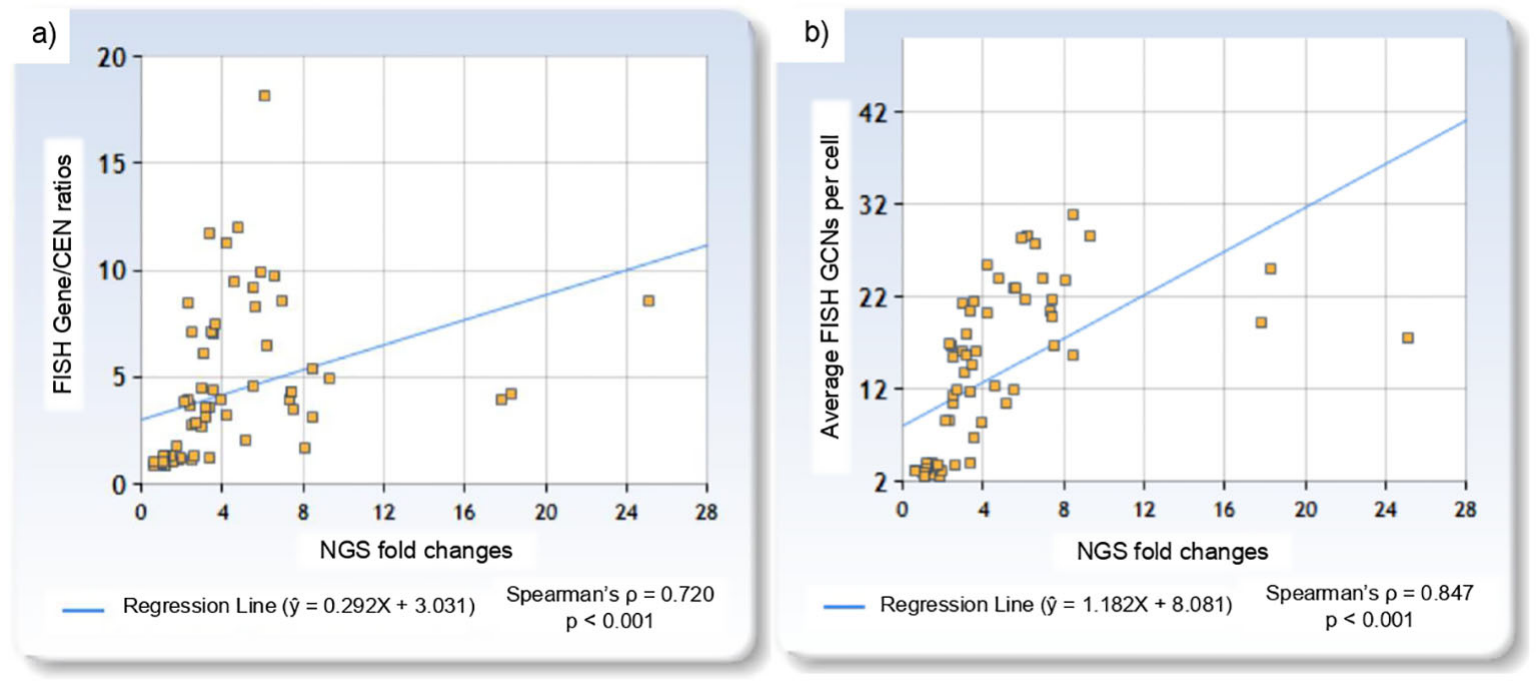
Linear regression plots showing: **(a)** the relationship between NGS fold changes and FISH Gene/CEN ratios, and **(b)** the relationship between NGS fold changes and gene copy number per cell.

**Figure 3:**
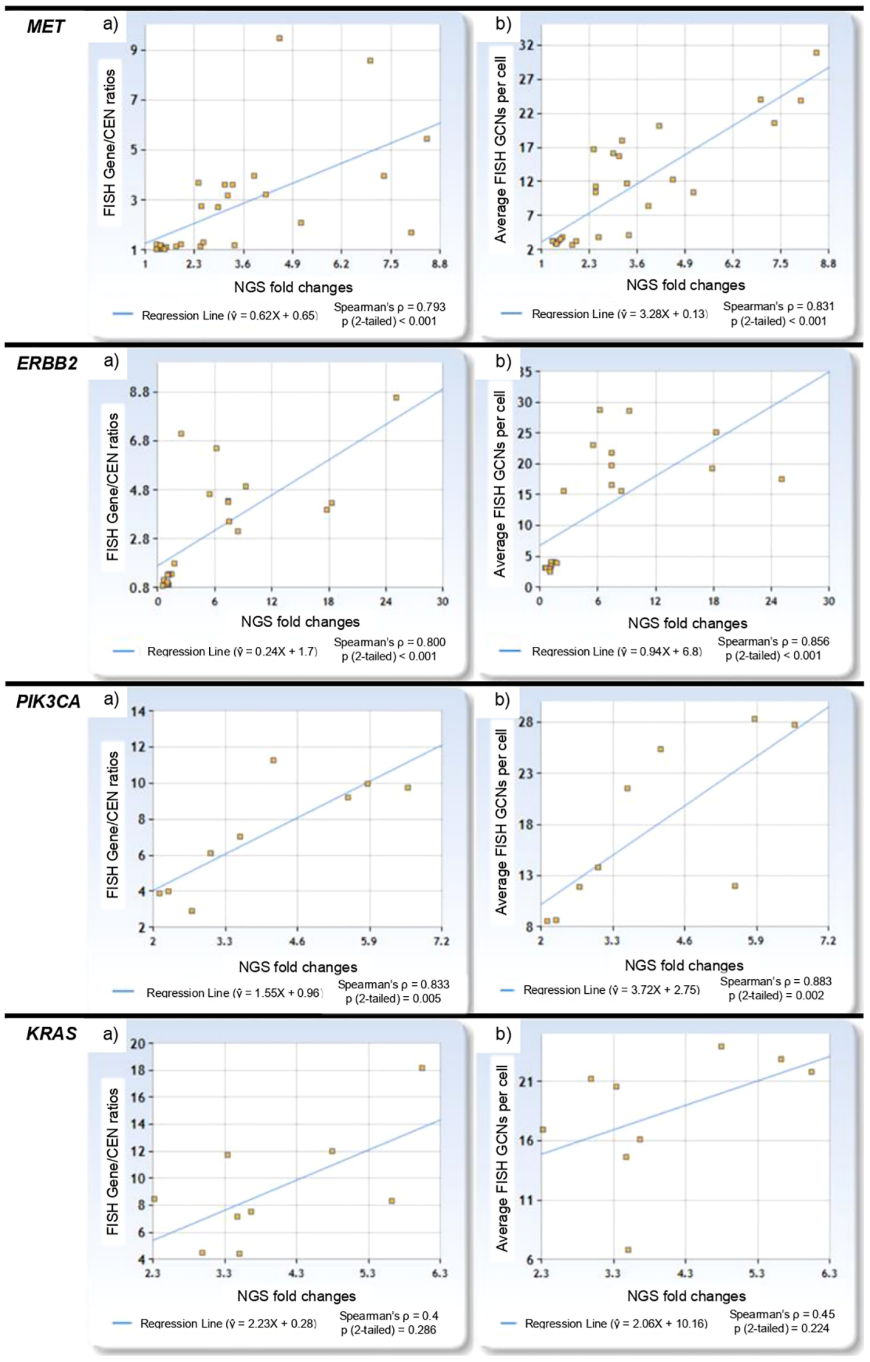
Linear regression plots for *MET*, *ERBB2*, *PIK3CA* and *KRAS* showing: **(a)** the relationship between NGS fold changes and FISH Gene/CEN ratios, and **(b)** the relationship between NGS fold changes and gene copy number per cell.

Among FISH-negative cases, NGS fold change ranged from 0.57 for *ERBB2* to 1.95 for *MET*, while in FISH-positive cases, it ranged from 2.11 for *PIK3CA* to 25.08 for *ERBB2*. Two *MET* cases with low level amplification had corresponding NGS fold changes of 2.54 and 3.36. NGS fold changes showed a positive correlation with FISH Gene/CEN ratios (Spearman’s ρ = 0.720, p < 0.001), with the linear regression model: ŷ = 0.292X + 3.031 (**Figure 2a**). Similarly, a positive correlation was observed between NGS fold changes and gene copy number per cell (Spearman’s ρ = 0.847, p < 0.001), described by the model: ŷ = 1.182X + 8.081 (**Figure 2b**). The linear regression plots for each gene are presented in **Figure 3**.

## 4 Discussion

FISH is still the gold standard for detecting gene amplifications and mainly used in clinical trials (9). This technique is particularly advantageous in cases with low TCC, tumor heterogeneity, and focal amplifications, as it allows for slide-based evaluation under a microscope (14, 21–23). Nonetheless, it also has limitations. The evaluation is observer-dependent, and tissue sectioning artefacts can impact the analysis. Additionally, a new slide of material is required for each additional parameter tested by FISH, which can be challenging when dealing with small biopsies (21, 22).

Our study demonstrates the potential of NGS as a reliable tool for identifying cases with gene amplifications in NSCLC, offering a high-throughput, scalable alternative to traditional FISH. While FISH remains the gold standard due to its high resolution and specificity, it can become impractical when multiple markers need to be tested, underscoring the need for more comprehensive approaches like NGS.

### 4.1 NGS as a predictive tool for gene amplification

The strong positive correlations observed between NGS fold changes and both FISH Gene/CEN ratios and gene copy number per cell underscore the utility of NGS in evaluating gene amplification status. This suggests that NGS could reliably indicate amplification without requiring confirmatory FISH, especially when fold changes are markedly elevated. For setting a cutoff to distinguish FISH-positive from FISH-negative cases based on NGS fold change, our results suggest that values below 2.0 tend to correspond with FISH-negative findings, while values above 2.0 align with FISH-positive results.

A challenge arises when FISH shows a low Gene/CEN ratio but a high average GCN. These cases could indicate true amplifications or high polysomy, yet may not exhibit a corresponding increase in NGS coverage (24). Visually identifying such cases by coverage alone can be challenging, as NGS might not show the expected increase in coverage. This is because NGS coverage reflects the total number of sequence reads mapped to a gene’s target regions, and if these regions are limited, subtle increases in gene copies might not significantly impact overall coverage (10). Moreover, technical factors such as TCC, sequencing depth, and the specific regions of the gene covered by the NGS panel can also influence the observed fold change. For instance, if a gene has multiple copies in a small percentage of tumor cells, the overall increase in coverage should be diluted by non-tumor cells, leading to an underestimation of amplification. In our study, we encountered 20 cases with low tumor cellularity (≤20%), which could potentially affect the accuracy of NGS-based copy number assessments. Although our analysis did not reveal significant discrepancies between NGS and FISH results in these cases, we acknowledge that low tumor cellularity is a known factor that can lead to underestimation of copy number amplification. This underscores the importance of considering tumor cellularity when interpreting NGS results and highlights the need for integrating multiple diagnostic modalities. Therefore, relying solely on NGS coverage as a predictive tool requires further validation on larger cohorts, along with more refined analytical methods and expanded gene coverage in NGS panels to enhance detection accuracy (25, 26). Additionally, assessing more genes with FISH is essential to determine if a 2.0 cutoff is universally optimal across various genes.

### 4.2 NGS panel and reference sequence

Our NGS panel focused on selected exons of particular interest, which could influence both coverage and correlation results. By targeting specific exons, the panel might not fully represent the gene’s overall amplification status, potentially leading to underestimation or overestimation of gene amplification (25, 26). This limitation is particularly relevant when the regions selected do not encompass the most critical areas of amplification across the entire gene. This selective coverage may also contribute to the variability observed in the regression slopes across different genes, as illustrated in **Figure 3**. For example, genes with exons more susceptible to technical factors—such as variations in probe efficiency or sequencing depth—might exhibit inconsistent fold changes compared to FISH results. Furthermore, the use of a single reference sequence for each gene in NGS adds another layer of variability to fold change calculations. Discrepancies between the reference sequence and the actual genomic sequence in the tumor samples could introduce inaccuracies in fold change measurements, affecting the reliability of NGS in predicting gene amplifications.

### 4.3 Future prospects

Integrating NGS into clinical diagnostics offers significant potential for advancing the detection and characterization of genomic alterations in cancer. Unlike FISH, which is restricted to specific probes and lower throughput, NGS can simultaneously detect small variants, GCN changes across numerous genes, and other key biomarkers such as tumor mutational burden (TMB), microsatellite instability (MSI), and homologous recombination deficiency (HRD) (27–30). This comprehensive capability makes NGS an invaluable tool for personalized cancer treatment, offering a more complete genomic profile with less observer dependence and reduced risk of tissue sectioning artifacts.

However, to fully harness the potential of NGS, further research is required to refine predictive models and establish standardized thresholds for NGS fold changes. While our study demonstrates the utility of NGS in detecting *MET* and *ERBB2* amplifications in NSCLC, the absence of *EGFR* amplifications in our cohort may potentially limit the generalizability of our findings. This absence reflects our sample selection strategy, which prioritized cases with other alterations rather than a cross-section of NSCLC cases. Future studies should include a broader range of gene amplifications, including *EGFR*, to comprehensively evaluate the performance of NGS across the diverse molecular landscape of NSCLC. Similarly, this study lacks non-amplified *PIK3CA* and *KRAS* cases that probably results in higher regression lines (**Figure 3**). A robust validation cohort should include not only cases with clear amplification and FISH-negative samples, but also low-coverage cases, and those with high GCNs but low Gene/CEN ratios. This would allow for the determination of a reliable cut-off for negative results and improve the specificity of NGS. Additionally, examining cases with amplification in the context of high polysomy could provide insights into how NGS distinguishes these from true gene amplifications. Notably, the amplification status in our study was determined based on established FISH criteria, which account for both Gene/CEN ratios and average GCNs. While this approach effectively identifies FISH-positive cases, it does not exclude the possibility of high polysomy being classified as amplification. Future analyses should explore this distinction further to enhance the accuracy of NGS-based classifications.

To further enhance the accuracy of NGS, expanding the panel to cover more regions in genes of interest could improve detection sensitivity. Additionally, in future studies, reporting the range of Gene/CEN ratios and GCNs for individual cells could provide valuable insights into intratumor heterogeneity and help further explore the correlation between NGS and FISH results. Refining this aspect could make NGS reliable alternative to FISH in routine diagnostics, particularly for cases where multiple biomarkers need to be assessed simultaneously.

## 5 Conclusions

NGS fold changes correlate well with both FISH Gene/CEN ratios and gene copy number per cell, supporting its potential as a marker for gene amplification. We established an NGS fold change cutoff of 2.0, effectively distinguishing amplified from non-amplified cases. While NGS provides a more efficient and comprehensive approach to cancer diagnostics by enabling the analysis of multiple genomic alterations in a single assay, further research addressing the limitations of this study will be essential to fully optimize NGS for routine diagnostic use.

## Data Availability

The datasets presented in this study can be found in online repositories. The names of the repository/repositories and accession number(s) can be found in the article/**Supplementary Material**.
