# Supplementary material for: NGS coverage accurately predicts MET and HER2 (ERBB2) gene amplifications in a real-world non-small cell lung cancer cohort
Source: Front Oncol. 2025 Jul 29;15:1618509. doi: 10.3389/fonc.2025.1618509 (PMC12340238; doi:10.3389/fonc.2025.1618509)
Supplement: Supplementary file 1 [file Table1.docx]

| **Gen** | **NM_Nummer** | **Exons** |
| --- | --- | --- |
| *ALK* | NM_004304 | 21-25 |
| *BRAF* | NM_004333 | 11, 15 |
| *CTNNB1* | NM_001904 | 3 |
| *EGFR* | NM_005228 | 18-21 |
| *ERBB2* | NM_004448 | 8, 19, 20 |
| *FGFR1* | NM_023110 | 4-7, 10, 12-15 |
| *FGFR2* | NM_000141 | 6 - 15, 18 |
| *FGFR2* | NM_022970 | 8 |
| *FGFR3* | NM_000142 | 3, 6, 7, 9, 10, 12 - 16, 18 |
| *FGFR4* | NM_213647 | 3, 6, 9, 12, 13, 15, 16 (Codon 672-712) |
| *HRAS* | NM_001130442 | 2-4 |
| *IDH1* | NM_005896 | 4 |
| *IDH2* | NM_002168 | 4 |
| *KEAP1* | NM_203500 | 2-6 |
| *KRAS* | NM_033360 | 2-4 |
| *MAP2K1* | NM_002755 | 2, 3 |
| *MET* | NM_001127500 | 14, 16 - 19 |
| *NRAS* | NM_002524 | 2-4 |
| *NTRK1* | NM_002529 | 13-17 |
| *NTRK2* | NM_006180 | 16-19 |
| *NTRK3* | NM_001012338 | 15-20 |
| *PIK3CA* | NM_006218 | 8, 10, 21 |
| *PTEN* | NM_000314 | 1-8 |
| *RET* | NM_020975 | 10-18 |
| *ROS1* | NM_002944 | 34-41 |
| *STK11* | NM_000455 | 1-9 |
| *TP53* | NM_000546 | 2-11 |

**Table S1. Gene Panel Details for NGS Analysis.**
