# Supplementary material for: NGS coverage accurately predicts MET and HER2 (ERBB2) gene amplifications in a real-world non-small cell lung cancer cohort
Source: Front Oncol. 2025 Jul 29;15:1618509. doi: 10.3389/fonc.2025.1618509 (PMC12340238; doi:10.3389/fonc.2025.1618509)
Supplement: Supplementary file 2 [file Table2.docx]

**Table S2. Characteristics of the samples collective including the NGS and FISH results.**

|  | **NGS** | | | | | **FISH** | | | | | |
| --- | --- | --- | --- | --- | --- | --- | --- | --- | --- | --- | --- |
|  |  |  |  |  |  |  |  |  |  |  |  |
|  | **Gene** | **TCC (%)** | **Mean coverage** | **Max. coverage for selected gene** |  | **Result** | **Gene Signals** | **CEN Signals** | **Gene/CEN** | **Polysomy (%)** | **Gene copies/cell** |
| **Sample ID** |  |  |  |  | **Fold change** |  |  |  |  |  |  |
| 1 | *MET* | 30 | 743.3 | 6282 | 8.45 | Positive: High | 619 | 114 | 5.43 | 75 | 30.95 |
| 2 |  | 15 | 1494.6 | 12013 | 8.04 | Positive: High | 478 | 279 | 1.71 | 100 | 23.9 |
| 3 |  | 20 | 577.8 | 4222 | 7.31 | Positive: High | 411 | 104 | 3.95 | 95 | 20.55 |
| 4 |  | 50 | 1687.9 | 11734 | 6.95 | Positive: High | 480 | 56 | 8.57 | 50 | 24 |
| 5 |  | 50 | 840.6 | 4309 | 5.13 | Positive: High | 208 | 99 | 2.1 | 90 | 10.4 |
| 6 |  | 50 | 1447.8 | 6604 | 4.56 | Positive: High | 246 | 26 | 9.46 | 0 | 12.3 |
| 7 |  | 40 | 1029 | 4319 | 4.2 | Positive: High | 404 | 126 | 3.21 | 100 | 20.2 |
| 8 |  | 20 | 990.5 | 3857 | 3.89 | Positive: High | 167 | 42 | 3.98 | 35 | 8.35 |
| 9 |  | 60 | 1127.3 | 3791 | 3.36 | Positive: Low | 248 | 206 | 1.2 | 68.33 | 4.13 |
| 10 |  | 70 | 1530 | 5072 | 3.32 | Positive: High | 235 | 65 | 3.62 | 65 | 11.75 |
| 11 |  | 20 | 1443.8 | 4590 | 3.18 | Positive: High | 360 | 114 | 3.16 | 90 | 18 |
| 12 |  | 40 | 824.5 | 2562 | 3.11 | Positive: High | 314 | 87 | 3.61 | 85 | 15.7 |
| 13 |  | 15 | 1150.6 | 3385 | 2.94 | Positive: High | 322 | 119 | 2.71 | 100 | 16.1 |
| 14 |  | 80 | 2224.2 | 5639 | 2.54 | Positive: Low | 228 | 176 | 1.3 | 56.67 | 3.8 |
| 15 |  | 20 | 2996.3 | 7416 | 2.48 | Positive: High | 225 | 82 | 2.74 | 90 | 11.25 |
| 16 |  | 70 | 1385.9 | 3422 | 2.47 | Positive: High | 209 | 181 | 1.15 | 100 | 10.45 |
| 17 |  | 30 | 1389.4 | 3352 | 2.41 | Positive: High | 335 | 91 | 3.68 | 90 | 16.75 |
| 18 |  | 65 | 1115.5 | 2174 | 1.95 | Negative | 64 | 52 | 1.23 | 55 | 3.2 |
| 19 |  | 20 | 523.2 | 955 | 1.83 | Negative | 53 | 47 | 1.13 | 30 | 2.65 |
| 20 |  | 20 | 641.1 | 1008 | 1.57 | Negative | 77 | 69 | 1.12 | 70 | 3.85 |
| 21 |  | 20 | 619.7 | 942 | 1.52 | Negative | 71 | 68 | 1.04 | 80 | 3.55 |
| 22 |  | 10 | 627.6 | 917 | 1.46 | Negative | 66 | 62 | 1.06 | 70 | 3.3 |
| 23 |  | 40 | 1579.9 | 2241 | 1.42 | Negative | 61 | 54 | 1.13 | 40 | 3.05 |
| 24 |  | 70 | 537.8 | 751 | 1.4 | Negative | 56 | 47 | 1.19 | 30 | 2.8 |
| 25 |  | 60 | 1233.1 | 1705 | 1.38 | Negative | 58 | 56 | 1.04 | 50 | 2.9 |
| 26 |  | 20 | 1372.8 | 1805 | 1.31 | Negative | 64 | 53 | 1.21 | 40 | 3.2 |
| 27 |  | 20 | 1281 | 1649 | 1.29 | Negative | 65 | 64 | 1.02 | 65 | 3.25 |
| 28 | *ERBB2* | 30 | 2777.7 | 69663 | 25.08 | Positive | 352 | 41 | 8.59 | 20 | 17.6 |
| 29 |  | 40 | 1626.6 | 29772 | 18.3 | Positive | 501 | 118 | 4.25 | 95 | 25.05 |
| 30 |  | 20 | 1644.1 | 29316 | 17.83 | Positive | 384 | 96 | 4 | 90 | 19.2 |
| 31 |  | 15 | 704 | 6541 | 9.29 | Positive | 573 | 116 | 4.94 | 90 | 28.65 |
| 32 |  | 40 | 744.7 | 6287 | 8.44 | Positive | 313 | 101 | 3.1 | 100 | 15.65 |
| 33 |  | 25 | 1964 | 14699 | 7.48 | Positive | 333 | 95 | 3.51 | 85 | 16.65 |
| 34 |  | 30 | 1503.4 | 11166 | 7.43 | Positive | 435 | 101 | 4.31 | 100 | 21.75 |
| 35 |  | 60 | 1127.3 | 8329 | 7.39 | Positive | 396 | 91 | 4.35 | 80 | 19.8 |
| 36 |  | 20 | 846.2 | 5256 | 6.21 | Positive | 574 | 88 | 6.52 | 100 | 28.7 |
| 37 |  | 70 | 1152.9 | 6352 | 5.51 | Positive | 461 | 100 | 4.61 | 80 | 23.05 |
| 38 |  | 60 | 1031.4 | 2547 | 2.47 | Positive | 312 | 44 | 7.09 | 25 | 15.6 |
| 39 |  | 30 | 880.3 | 1561 | 1.77 | Negative | 234 | 132 | 1.77 | 32 | 3.9 |
| 40 |  | 40 | 2700.9 | 3971 | 1.47 | Negative | 244 | 180 | 1.36 | 61.67 | 4.07 |
| 41 |  | 38 | 446.3 | 520 | 1.17 | Negative | 80 | 87 | 0.92 | 85 | 4 |
| 42 |  | 35 | 2031.7 | 2349 | 1.16 | Negative | 74 | 56 | 1.32 | 55 | 3.7 |
| 43 |  | 40 | 570.6 | 655 | 1.15 | Negative | 76 | 57 | 1.33 | 55 | 3.8 |
| 44 |  | 30 | 363.6 | 418 | 1.15 | Negative | 70 | 77 | 0.91 | 100 | 3.5 |
| 45 |  | 20 | 1561.7 | 1633 | 1.05 | Negative | 60 | 46 | 1.3 | 40 | 3 |
| 46 |  | 20 | 258.6 | 266 | 1.03 | Negative | 51 | 49 | 1.04 | 45 | 2.55 |
| 47 |  | 30 | 294.4 | 299 | 1.02 | Negative | 59 | 63 | 0.94 | 65 | 2.95 |
| 48 |  | 50 | 2613 | 1569 | 0.6 | Negative | 62 | 57 | 1.09 | 55 | 3.1 |
| 49 |  | 40 | 2019 | 1158 | 0.57 | Negative | 63 | 73 | 0.86 | 90 | 3.15 |
| 50 | *PIK3CA* | 60 | 1680.7 | 11069 | 6.59 | Positive | 555 | 57 | 9.74 | 50 | 27.75 |
| 51 |  | 30 | 1780.5 | 10431 | 5.86 | Positive | 567 | 57 | 9.95 | 65 | 28.35 |
| 52 |  | 80 | 1659.1 | 9127 | 5.5 | Positive | 239 | 26 | 9.19 | 0 | 11.95 |
| 53 |  | 25 | 1238.9 | 5160 | 4.16 | Positive | 508 | 45 | 11.29 | 30 | 25.4 |
| 54 |  | 25 | 1811.6 | 6441 | 3.56 | Positive | 430 | 61 | 7.05 | 60 | 21.5 |
| 55 |  | 20 | 1071.7 | 3255 | 3.04 | Positive | 276 | 45 | 6.13 | 25 | 13.8 |
| 56 |  | 60 | 1353.5 | 3647 | 2.69 | Positive | 238 | 82 | 2.9 | 75 | 11.9 |
| 57 |  | 15 | 501.1 | 1145 | 2.28 | Positive | 172 | 43 | 4 | 30 | 8.6 |
| 58 |  | 40 | 2700.9 | 5710 | 2.11 | Positive | 171 | 44 | 3.89 | 25 | 8.55 |
| 59 | *KRAS* | 40 | 1536.9 | 9277 | 6.04 | Positive | 437 | 24 | 18.2 | 5 | 21.85 |
| 60 |  | 30 | 1786.4 | 10037 | 5.62 | Positive | 458 | 55 | 8.33 | 60 | 22.9 |
| 61 |  | 30 | 1306.5 | 6253 | 4.79 | Positive | 479 | 40 | 11.98 | 20 | 23.95 |
| 62 |  | 25 | 252.3 | 924 | 3.66 | Positive | 323 | 43 | 7.51 | 20 | 16.15 |
| 63 |  | 25 | 2088.1 | 7317 | 3.5 | Positive | 136 | 31 | 4.39 | 0 | 6.8 |
| 64 |  | 60 | 1532.9 | 5319 | 3.47 | Positive | 293 | 41 | 7.15 | 20 | 14.65 |
| 65 |  | 40 | 1021.1 | 3414 | 3.34 | Positive | 411 | 35 | 11.74 | 15 | 20.55 |
| 66 |  | 60 | 739.6 | 2213 | 2.99 | Positive | 425 | 95 | 4.47 | 5 | 21.25 |
| 67 |  | 20 | 209.6 | 486 | 2.32 | Positive | 339 | 40 | 8.48 | 5 | 16.95 |
| TCC, Tumor Cell Content; CEN, Centromere | | | | | | | | | | | |
